# Supplementary material for: Novel Insights for Patients with Multiple Basal Cell Carcinomas and Tumors at High-Risk for Recurrence: Risk Factors, Clinical Morphology, and Dermatoscopy
Source: Cancers (Basel). 2021 Jun 27;13(13):3208. doi: 10.3390/cancers13133208 (PMC8269292; doi:10.3390/cancers13133208)
Supplement: Supplementary file 1 [file cancers-13-03208-s001.zip › cancers-1261085-supplementary.pdf]

**Table S1. Definition of dermatoscopic terms and criteria[4, 19]**

| | |
|---|---|
| **Vasculature*** | The presence of vascular features |
| None / Apparent <50% / Prominent ≥50% (lesion's total surface) | |
| **Vessels** | Pink to Red Vascular structures |
| Arborizing | In-focused, bright red, tree-branched vessels |
| Telangiectasias | Short, fine linear vessels with very few branches |
| Glomerular | Tortuous vessels |
| Linear | Serpentine vessels irregular in shape and width |
| Dotted | Vessels as dots arranged fairly closely |
| Hairpin | Looped vessels |
| Polymorphous | A combination of ≥2 different types of vessels |
| **Erosions / Ulcerations*** | Loss of the epidermis presented as orange to black crusts |
| None / Erosions / Prominent <90% / >90% (lesion's total surface) | |
| **Pigmentation*** | The presence of pigmented structures |

| | |
|---|---|
| None / Light <10% / Partial 10-50% / Heavy >50% (lesion's total surface) | |
| Blue-gray ovoid nests/globules | Ovoid, well-circumscribed, plump blue-gray globular structures |
| Blue-gray dots | Pin-point closely arranged blue-gray dots |
| Spoke-wheel structures | Well-circumscribed radial projections meeting at a darker globular center (light to dark brown/black/blue-gray |
| Leaf-like structures | Peripheral light brown to bluish bulbous projections resembling maple leaf |

**Table S2. Solitary and multiple BCC tumors characteristics, n=304**

| | **Total (n=304)** | **Solitary (n=172)** | **Multiple (n=132)** |
|---|---|---|---|
| **Number of patients, *n=225* (%)** | 225 (100) | 172 (76.4) | 53 (23.6) |
| **Number of BCC, n(%)** | 304 (100) | 172 (56.6) | 132 (43.4) |
| *Number of tumors per individual, n=53(%)* | | | |
| *2* | | | 38 (71.7) |
| *3* | | | 9 (17.1) |
| *4* | | | 3 (5.6) |
| *≥5* | | | 3 (5.6) |
| **Anatomic location, n(%)** | | | |
| Head/neck | 223 (73.4) | 128 (74.6) | 95 (72) |
| Trunk | 58 (19.1) | 33 (19.2) | 25 (18.9) |
| Extremities | 23 (7.5) | 11 (6.4) | 12 (9.1) |
| **Diameter, median (range) cm** | 0.9 (0.2 – 12.3) | 0.95 (0.3-12.3) | 0.85 (0.2-7.2) |
| **Risk for local recurrence & metastasis, n(%)** | | | |
| Low-risk | 92 (30.3) | 46 (26.7) | 46 (34.8) |
| High-risk | 212 (69.7) | 126 (73.3) | 86 (65.2) |
| **Histopathology** | | | |

| | | | |
|---|---|---|---|
| **Indolent growth pattern, n(%)** | 224 (73.7) | 122 (70.9) | 102 (77.3) |
| Nodular | 175 (78.1) | 96 (78.7) | 79 (77.4) |
| Superficial | 49 (21.9) | 26 (21.3) | 23 (22.6) |
| **Aggressive growth pattern, n(%)** | 80 (26.3) | 50 (29.1) | 30 (22.7) |
| Infiltrative | 42 (13.8) | 25 (14.5) | 17 (12.9) |
| Morpheiform | 1 (0.3) | 1 (0.6) | 0 |
| Basosquamous | 14 (4.6) | 10 (5.8) | 4 (3) |
| Micro-nodular | 2 (0.7) | 0 | 2 (1.5) |
| Mixed | 21 (6.9) | 14 (8.1) | 7 (5.3) |

**Table S3. Univariate logistic regression for the clinical features of high-risk Vs. low-risk BCCs**

| | **p-value** | **OR** | **95% CIs** |
|---|---|---|---|
| **Margins** | <0.001 | 3.514 | 1.867-6.615 |
| **Ulceration** | | | |
| Erosions Vs none | 0.33 | | |
| Prominent Vs none | <0.001 | 3.782 | 2.039-7.015 |
| >90% vs none | <0.001 | 14.184 | 3.237-62.153 |
| **Pink color** | 0.417 | | |
| **Clinical Presentation** | | | |
| Elevated vs flat | 0.001 | 4.5 | 1.92-10.546 |
| Nodular vs flat | <0.001 | 5.708 | 2.633-12.375 |
| **White color** | <0.001 | 3.067 | 1.809-5.202 |
| **Blue-black color** | 0.001 | 0.433 | 0.262-0.715 |
| **Pigmentation** | | | |
| Light Vs none | 0.006 | 0.38 | 0.192-0.752 |
| Partial vs none | 0.032 | 0.443 | 0.211-0.933 |
| Heavy vs none | 0.31 | | |

**Table S4. Multivariate logistic regression analysis for the clinical features of high-risk Vs. low-risk BCCs**

| | p-value | OR | 95% CIs |
|---|---|---|---|
| **Margins** | 0.067 | 2.007 | 0.952-4.23 |
| **Ulceration** | | | |
| Erosions Vs none | 0.354 | | |
| Prominent Vs none | 0.011 | 2.533 | 1.243-5.162 |
| >90% vs none | 0.008 | 9.241 | 1.79-47.711 |
| **Clinical Presentation** | | | |
| Elevated vs flat | 0.083 | 2.384 | 0.892-6.376 |
| Nodular vs flat | 0.004 | 3.674 | 1.502-8.988 |
| **White color** | <0.001 | 3.682 | 1.988-6.819 |
| **Blue-black color** | 0.071 | 0.193 | 0.032-1.153 |
| **Pigmentation** | | | |
| Light Vs none | 0.504 | | |
| Partial vs none | 0.657 | | |
| Heavy vs none | 0.088 | 5.611 | 0.771-40.82 |

For the final model a fitness of good control was performed based on Hosmer-Lemeshow criterion (p-value=0.547)

**Table S5. Univariate logistic regression analysis for the dermatoscopic features of high-risk Vs. low-risk BCCs**

| | p-value | OR | 95% CIs |
|---|---|---|---|
| **Vasculature** | 0.394 | | |
| **Vessels** | | | |
| Arborizing | 0.002 | 2.52 | 1.394-4.555 |
| Telangiectasias | 0.005 | 0.464 | 0.273-0.789 |
| Glomerular | 0.008 | 4.24 | 1.446-12.449 |
| Linear irregular | 0.115 | | |
| Dotted | N/A | | |
| Hairpin | N/A | | |
| Polymorphous | N/A | | |
| **Pigmented structures** | | | |
| Blue-gray ovoid globules | 0.056 | 0.618 | 0.377-1.013 |
| Multiple Dots | 0.001 | 0.401 | 0.239-0.675 |

| | | | |
|---|---|---|---|
| Spoke-Wheel | 0.001 | 0.228 | 0.096-0.542 |
| Leaf-like | <0.001 | 0.204 | 0.09-0.461 |
| Concentric | 0.007 | 0.239 | 0.084-0.678 |
| **Pigmentation intensity** | | | |
| Light | <0.001 | 0.301 | 0.162-0.561 |
| Partial | 0.002 | 0.301 | 0.143-0.635 |
| Heavy | 0.032 | 0.437 | 0.205-0.93 |
| **Pink-whitish background** | 0.054 | 0.575 | 0.327-1.011 |
| **Diffuse white color** | 0.096 | 5.816 | 0.731-46.252 |
| **White shiny lines** | 0.002 | 2.304 | 1.352-3.925 |
| **Multiple yellow-white globules** | 0.193 | | |
| **White circles & Yellow Clods** | 0.038 | 3.199 | 1.065-9.605 |
| **Ulceration** | | | |
| Erosions vs none | 0.8 | | |
| Prominent vs none | <0.001 | 3.055 | 1.635-5.707 |
| >90% vs. none | 0.001 | 12.33 | 2.759-55.104 |

**Table S6. Multivariate logistic regression for the dermatoscopic features of high-risk Vs. low-risk BCCs**

| | p-value | OR | 95% CIs |
|---|---|---|---|
| **Vessels** | | | |
| Glomerular | 0.044 | 3.314 | 1.033-10.626 |
| Linear irregular | 0.195 | | |
| **Pigmentation intensity** | | | |
| Light | <0.001 | 0.269 | 0.13-0.558 |
| Partial | 0.001 | 0.198 | 0.078-0.5 |
| Heavy | 0.037 | 0.313 | 0.105-0.934 |
| **Pink-whitish background** | 0.021 | 0.369 | 0.158-0.862 |
| **Diffuse white color** | 0.128 | | |
| **White shiny lines** | 0.025 | 2.087 | 1.097-3.971 |
| **White circles & Yellow Clods** | 0.154 | | |
| **Ulceration** | | | |
| Erosions vs none | 0.944 | | |
| Prominent vs none | 0.014 | 2.451 | 1.198-5.014 |
| >90% vs. none | 0.01 | 8.042 | 1.637-39.505 |

**Table S7. Dermatoscopic findings in solitary and multiple BCC**

| | **Total (n=304)** | **Solitary (n=172)** | **Multiple (n=132)** |
|---|---|---|---|
| **Vasculature, n (%)** | | | |
| None | 32 (10.6) | 21 (12.2) | 11 (8.3) |
| Apparent | 219 (72) | 110 (64) | 109 (82.6) |
| Prominent | 53 (17.4) | 41 (23.8) | 12 (9.1) |
| **Vessels, n (%)** | | | |
| Arborizing | 247 (81.3) | 138 (80.2) | 109 (82.6) |
| Telangiectasias | 82 (27) | 49 (28.5) | 33 (25) |
| Glomerular | 34 (11.2) | 18 (10.4) | 16 (12.1) |
| Linear irregular | 14 (4.6) | 7 (4.1) | 7 (5.3) |
| Dotted | 1 (0.3) | 1 (0.6) | 0 |
| Hairpin | 3 (1) | 3 (1.7) | 0 |
| Polymorphous | 14 (4.6) | 10 (5.8) | 4 (3) |
| **Pigmented structures, n (%)** | | | |
| Blue-gray ovoid globules | 127 (41.8) | 72 (41.9) | 55 (41.7) |
| Multiple Dots | 90 (29.6) | 50 (29.1) | 40 (30.3) |
| Spoke-Wheel | 24 (7.9) | 12 (7) | 12 (9.1) |
| Leaf-like | 28 (9.2) | 16 (9.3) | 12 (9.1) |
| Concentric | 16 (5.3) | 12 (7) | 4 (3) |
| **Pigmentation intensity, n (%)** | | | |
| None | 141 (46.4) | 82 (47.7) | 59 (44.7) |
| Light | 77 (25.3) | 41 (23.8) | 36 (27.3) |
| Partial | 42 (13.8) | 26 (15.1) | 16 (12.1) |
| Heavy | 44 (14.5) | 23 (13.4) | 21 (15.9) |
| **Pink-whitish background, n (%)** | 211 (69.4) | 120 (69.8) | 91 (68.9) |
| **Diffuse white color, n (%)** | 11 (3.6) | 7 (4.1) | 4 (3) |
| **White shiny lines, n (%)** | 123 (40.5) | 89 (51.7) | 34 (25.8) |
| **Multiple yellow-white globules, n (%)** | 34 (11.2) | 28 (16.3) | 6 (4.6) |
| **White circles & Yellow Clods, n(%)** | 62 (20.4) | 42 (24.4) | 19 (14.4) |
| **Ulceration, n (%)** | | | |
| None | 79 (26) | 40 (23.2) | 39 (29.6) |

| | | | |
|---|---|---|---|
| Erosions | 71 (23.3) | 40 (23.3) | 31 (23.5) |
| Prominent | 121 (39.8) | 69 (40.1) | 52 (39.4) |
| >90% | 33 (10.9) | 23 (13.4) | 10 (7.5) |

**Table S8. Dermatoscopic features in histologically aggressive subtypes of BCC (n=80)**

| | **Aggressive histologic subtypes of BCC (n=80)** | **High-Risk (n=212)** |
|---|---|---|
| **Vasculature, n (%)** | | |
| None | 9 (11.3) | 19 (9) |
| Apparent (<50%) | 60 (75) | 156 (73.6) |
| Prominent (≥50%) | 11 (13.7) | 37 (17.4) |
| **Vessels, n (%)** | | |
| Arborizing | 69 (86.3) | 182 (85.9) |
| Telangiectasias | 23 (28.7) | 47 (22.2) |
| Glomerular | 15 (18.7) | 30 (14.2) |
| Linear irregular | 8 (10) | 12 (5.7) |
| Dotted | 1 (1.3) | 1 (0.5) |
| Hairpin | 1 (1.3) | 3 (1.4) |
| Polymorphous | 9 (11.3) | 14 (6.6) |
| **Pigmented structures, n (%)** | | |
| Blue-gray ovoid globules | 23 (28.8) | 81 (38.2) |
| Multiple Dots | 15 (18.8) | 50 (23.6) |
| Spoke-Wheel | 0 | 9 (4.2) |
| Leaf-like | 0 | 10 (4.7) |
| Concentric | 0 | 6 (2.8) |
| **Pigmentation intensity, n (%)** | | |
| None | 49 (61.3) | 115 (54.3) |
| Light (<10%) | 18 (22.5) | 44 (20.8) |
| Partial (10%-50%) | 5 (6.25) | 24 (11.3) |
| Heavy (>50%) | 8 (10) | 29 (13.7) |
| **Pink-whitish background, n (%)** | 50 (62.5) | 140 (66) |
| **Diffuse white color, n (%)** | 8 (10) | 10 (4.7) |

| | | |
|---|---|---|
| **White shiny lines, n (%)** | 45 (56.3) | 98 (46.2) |
| **Multiple yellow-white globules, n (%)** | 11 (13.8) | 27 (12.7) |
| **White circles & Yellow Clods, n(%)** | 24 (30) | 55 (25.9) |
| **Ulceration, n (%)** | | |
| None | 10 (12.5) | 44 (20.8) |
| Erosions | 12 (15) | 41 (19.3) |
| Prominent | 37 (46.3) | 96 (45.3) |
| >90% | 21 (26.2) | 31 (14.6) |

**Table S9. Aggressive histologic subtypes of BCC, n=80 (%)**

| Infiltrative | Morpheaform | Basosquamous | Micro-nodular | Mixed |
|---|---|---|---|---|
| 42 (52.5%) | 1 (1.3%) | 14 (17.5) | 2 (2.5%) | 21 (26.3%) |
